# Data for sale: trust, confidence and sharing health data with commercial companies

**DOI:** 10.1136/medethics-2021-107464

**Published:** 2021-07-30

**Authors:** Mackenzie Graham

**Affiliations:** Wellcome Centre for Ethics and Humanities, University of Oxford, Oxford, UK

**Keywords:** ethics, information technology, confidentiality/privacy, databases- genetic, ethics- research

## Abstract

Powered by ‘big health data’ and enormous gains in computing power, artificial intelligence and related technologies are already changing the healthcare landscape. Harnessing the potential of these technologies will necessitate partnerships between health institutions and commercial companies, particularly as it relates to sharing health data. The need for commercial companies to be trustworthy users of data has been argued to be critical to the success of this endeavour. I argue that this approach is mistaken. Our interactions with commercial companies need not, and should not, be based on trust. Rather, they should be based on confidence. I begin by elucidating the differences between trust, reliability, and confidence, and argue that trust is not the appropriate attitude to adopt when it comes to sharing data with commercial companies. I argue that what we really should want is confidence in a system of data sharing. I then provide an outline of what a confidence-worthy system of data sharing with commercial companies might look like, and conclude with some remarks about the role of trust within this system.

## Introduction

Collections of health data, such as that currently held by the UK’s National Health Service (NHS), have been lauded as having the potential to ‘transform patient care’.^1^ Once curated, these datasets can be used to develop artificial intelligence and related technologies that can better identify risk factors and detect early signs of disease, guide therapy and management, and facilitate the development of future drugs and interventions.

Health data also have potential value as a commercial asset. The value of the data held by the NHS has been estimated at as much as £5 billion per year, with a further £4.6 billion of benefit through cost savings and improved patient outcomes.^2^ Accordingly, there has been an effort by government to capitalise on the potential value of healthcare data through partnerships with commercial companies.^3 4^

At the same time, research in 2020 found that less than 25% of UK respondents would be willing to share their anonymised medical data with a for-profit company.^5 6^ This figure represents a sharp decline from earlier research in 2016, which found that 59% of UK respondents supported sharing their data with commercial companies undertaking health research.^7^

For this reason, securing the trust of the public is often presented as an imperative for ethical data sharing.^8–12^ For example, guidelines published by the UK government highlight the need to approach the use of health data ‘in a way that is conducive to public trust’.^12^ Similarly, the Academy of Medical Sciences recommends as a core principle of data-driven technologies that they ‘maintain trustworthiness in the responsible and effective stewardship of patient data within the NHS’.”^13^

Yet, the importance placed on trust and trustworthiness belies the precision with which these concepts are typically used. This can make it difficult to identify and address the ethical problems raised in situations that ostensibly involve trust. Indeed, as I will argue, once we clearly articulate the concept, it turns out that trust is not the appropriate attitude to adopt in the context of data sharing with commercial companies. Instead, I propose that data sharing with commercial companies should be worthy of *confidence*.

## Reliability, trust and confidence

### Trust and reliance

Most philosophical accounts of trust adopt two basic conditions. First, trust is primarily a relation involving two people and a task. I trust my neighbour to walk my dog, my friend to keep a secret or my doctor to act in the best interests of my health. Second, trust involves expectations about both competence and willingness. If I trust my neighbour to take care of my dog while I am on vacation, I believe that she has the necessary skills and is willing to exercise them as required.^14^ Trusting another person is risky because it involves uncertainty about how the other person will act. If there were some guarantee or assurance against failure, there would be no need for trust. Trust therefore requires that we can be vulnerable to others; without the possibility of betrayal (or at least disappointment), there is no trust.

Trust can be distinguished from mere reliance. To rely on someone or something to X simply involves acting as if X will occur.^15^ Thus, a reliable person or object is one that behaves predictably. I might rely on my alarm clock to wake me up in the morning, or my partner to buy milk on her way home from work. Trust, on the other hand, is more than mere reliance; it includes an ‘extra factor’ that accounts for why the trusted person is being relied on. This extra factor might be a belief about the motives or goodwill of the trusted person,^16–18^ a belief that the trusted person has made a commitment^14 19^ or that the trusted person is responsive to the normative expectations of the trusting person.^20^ Suppose I trust you to give me a ride to an important meeting. In doing so, I am predicting that you will act in a certain way because you care about my interests (or have made a commitment, etc). Conversely, suppose I rely on the bus to take me to work, but it is late due to heavy traffic. It would be inappropriate to feel betrayed or demand an apology of the bus driver; this is a failure of reliance, not of trust.

### Trustworthiness

Closely related to the concept of trust is trustworthiness. Someone is trustworthy when our trust in them is well-grounded. Ideally, those whom we trust will also be trustworthy and those who are trustworthy will also be trusted. Of course, this is not always the case. This is because trustworthiness depends on features of the object of trust, while trusting depends on features of the person placing their trust. For example, suppose a company reliably disciplines employees guilty of sexual harassment, but only to avoid negative publicity. Such a company would not be trustworthy to promote a safe and equitable work environment, because it does not have the right motives (eg, care for its employees’ safety), or because it has not clearly committed to disciplining employees (ie, the discipline is contingent on avoiding bad publicity). Yet the employees might nevertheless trust the company to discipline cases of sexual harassment because they (mistakenly) believe that the company is motivated to provide a safe work environment.

### Confidence

The idea of confidence is rarely mentioned in the philosophical literature on trust and trustworthiness. However, it is an important part of the sociological literature on trust, most prominently in the work of Niklas Luhmann, Georg Simmel, and Anthony Giddens.^21–23^ Confidence is concerned with the social and technological systems within which people interact. These systems are based on generalised norms or mechanisms (eg, laws, regulations), the normal functioning of which leads to predictable outcomes. It is our beliefs about how these systems function, and thus, what can predictably be expected from our interactions with others within them, that allows confidence to form.^24 25^

Accordingly, confidence refers to something like ‘assured reliance’.^1^ When I rely on someone or something to X, I act as though X will happen, even though there are no assurances or guarantees that it will. Conversely, when I have confidence that X will happen, I act as though X will happen because I have some assurances or guarantees that it will happen. These assurances or guarantees are provided by the norms and mechanisms of the system in which X takes place, and do not require any personal knowledge about the person I am interacting with.

Suppose I need to hire a lawyer. Our interaction takes place within the context of a number of overlapping systems, each providing various assurances. It is my confidence in these systems that shapes my expectations for our interaction. Her law degree provides an assurance of her education, her certification to practice law assures her competence as a lawyer and the broader legal system guarantees me further legal rights, including the ability to impose formal sanctions if she fails to satisfy her obligations. This is more than mere reliance (I am not simply acting as though the lawyer will protect my legal interests), but it need not involve trust. I need not believe that the lawyer has the right kinds of motives or values, or that she has made a commitment to me (she may protect my interests purely to preserve her reputation, or be motivated entirely by money). Of course, I might eventually form these beliefs, in which case my confidence might be replaced by trust.

In the same way that an individual may or may not be trustworthy, my interactions within a system may be more or less worthy of confidence. A system is worthy of confidence when there are rules and norms sufficient to assure reliable interactions within it. If a system fails to yield predictable interactions, it is not confidence-worthy.

It is also possible to have confidence in a system that is not confidence-worthy, or to lack confidence in a confidence-worthy system. If I am not aware of its rules or norms, or have mistaken beliefs about their efficacy or reliability, I may not be confident in a system that is nevertheless worthy of confidence. For example, I may lack confidence in the pharmaceutical industry (and so be reluctant to receive a new vaccination), despite the fact that the safety of the vaccine is assured by stringent regulations. Similarly, if I falsely believe that there are rules or norms sufficient to ensure reliable outcomes, I might have confidence in a system that is not confidence-worthy. For example, I might rely on the results of a direct-to-consumer genetic test because I (mistakenly) have confidence that only reliable genetic tests would be available to consumers.

Confidence is thus distinct from trust. Whereas trust combines reliance with a belief about the internal motives or commitments of the trusted person, confidence combines reliance with a belief about the external norms and mechanisms of the system within which an interaction takes place. These norms and mechanisms reduce vulnerability by providing assurances or guarantees of certain outcomes, thereby obviating the need for trust. It is because of my confidence in the system of traffic rules that I drive through a green light without worrying about other cars hitting me, not my trust in other drivers. Conversely, when there is no basis for confidence—when our interactions take place outside of a system that provides guarantees or assurances of certain outcomes—we may need to trust. Indeed, in some cases, such as personal relationships, we may prefer our interactions to be based on trust rather than guarantees or assurances.

### Trust and trustworthiness in commercial companies

The sharing of health data occurs within a vast and complex network, involving different kinds of relationships. Patients might share their health information directly with their physician, research participants might share their information with researchers or biobanks, and de-identified or anonymised health information collected for care purposes may eventually be shared with governments, charities, or other organisations, including commercial companies. Our expectations for sharing our data depend on the nature of each of these relationships. My willingness to share my health data with my physician may be different from my willingness to share my health data with my local city council because there is a level of trust in the former relationship that does not exist in the latter. Accordingly, my expectations for how my data may permissibly be used may vary based on the nature of the relationship. For example, I may be happy for my physician to share health information about me with another physician for the purposes of my care, but would permit a researcher to share my data with other researchers only if it is de-identified, or only with my explicit consent. In the remainder of this paper, I will focus on one kind of ‘relationship’ that exists within the larger system of health data sharing: the relationship between individuals that are the source of health data, and the commercial companies that seek to use it. Should this relationship be one of trust, and if not, how should we understand it?

The first step is to identify the objects of possible trust. One possibility is the individual researchers using the data. Individual researchers have the capacity to affect my interests through their use of my data. By sharing it, I thereby make myself vulnerable to them, and rely on them to use my data appropriately. Have I also trusted them?

I suggest not. First, it is difficult to determine which individuals are being relied on, and for what purpose, even within a single company. Second, it is not always clear how to assign responsibility for the various ways in which data are used; is a particular researcher acting on their own or following company policy, for example? Third, without knowing how the motivations or commitments of individuals influence how data are actually used, it is hard to judge the basis for trust. This may be particularly true of large companies, where the motivations and values of the individual researcher may be different from those of the company. Each of these factors poses an obstacle not only to establishing trustworthiness, but also to trusting.

Accordingly, it seems that companies themselves, rather than the individuals that comprise them, would be the proper objects of trust. While most philosophical accounts of trust and reliance focus on individuals, these concepts can also be applied to groups and institutions.^19^ When we say that a group of people (eg, a company) is reliable, sometimes we mean that all or most of the individuals that comprise the group are reliable in a relevant way. In other cases, we mean that the group as a whole is reliable. I will focus on the latter conception of group reliability, and take for granted that it is possible for groups like companies to act reliably or unreliably in certain ways.^19^

If we are willing to grant that companies as a whole can be reliable or unreliable, we might also think that they can be the object of trust; this seems to be implied by the imperative to ‘trust companies with patient health data’, or the requirement that companies must ‘maintain trustworthiness’. What are they being trusted to do? Based on statements such as the one given by the UK Department of Health and Social Care, companies are being trusted to use health data in a way that protects the safety and security of those whose data are used, and ‘improves the health, welfare and/or care of patients’.^12^

I maintain that the relationship between individual contributors of health data and commercial companies need not, and should not, be based on trust. We need not willingly make ourselves vulnerable to them. First, commercial companies have legitimate interests and aims (eg, profits), which may conflict with the interests and aims of individuals that would trust them. In other words, companies may lack the kinds of motives and values—or the appropriate ordering of them—required to be trustworthy. Similarly, commercial companies have various commitments (eg, to shareholders) that shape their behaviours and practices, and those commitments may supersede the commitments to individuals that are necessary for trustworthiness.

Of course, the fact that companies have profit motives does not entail that these motives must always win out. Companies could choose to put considerations like societal benefit ahead of profits in some cases. Yet while some companies do place social benefit on equal footing with profitability, most do not. This is not to say that most companies purposely disregard social benefits in favour of profits, but rather that social benefits are in many cases not a priority.

Besides the potential for conflicting motives or commitments, it is not clear that commercial companies are competent to act in the best interests of individuals. They are not necessarily in a position to determine what is in the best interest of patient health or well-being, or that of society more generally. For example, the ethical problems created by inequitable or discriminatory algorithms in the healthcare context are well-established, despite presumably being intended to improve care.^26–28^ Even if a company has a ‘good will’ or beneficent motives or commitments, their conception of what is in the best interests of patients and society may be mistaken.

Sharing health data with commercial companies also has clear risks. Individuals are vulnerable to the improper use of their data, and potentially transformative technologies could exacerbate existing healthcare inequalities. Making data available to commercial companies also risks further concentrating power and influence over important societal concerns in the hands of large technology companies. At the same time, there are risks in *not* sharing patient data with commercial companies; they are a necessary part of healthcare innovation. Accordingly, given what is at stake, it is reasonable to require assurances about how health data will be used. The need for assurances, rather than a reliance on the motives or commitments of companies, suggests that trust is not appropriate.

Second, evaluating the trustworthiness of companies is more difficult than evaluating the trustworthiness of individuals, because it can be difficult to monitor their behaviour to the extent necessary to verify their motives or commitments. Because explicit consent is not always possible, or required, for data sharing, individuals have a limited ability to evaluate the trustworthiness of the particular companies that access it. It is unreasonable to expect individuals to understand and keep track of the myriad complex ways that different companies use their data, and restrict the sharing of their data to only those companies that are trustworthy. Indeed, unless individuals license their health data directly to companies, this kind of control is impossible.

Third, when it comes to sharing data with commercial companies, trust is merely a means to securing particular outcomes (eg, technologies to improve healthcare, economic growth, societal benefits). Provided these outcomes are realised, the motives and commitments of commercial companies in bringing them about are arguably less relevant. Contrast this with circumstances in which most would agree that trust is important: personal relationships. It may be important to me that I can trust my friend to keep a secret because trusting adds something valuable to our relationship.

Yet, it is not sufficient that companies be merely reliable. On the one hand, a company might be reliable in its *misuse* of patient data. We need companies to be reliable in the right sort of way. On the other hand, even if a company reliably brings about good outcomes now, there is no assurance that they will continue to do so. Further, the appropriate response to the misuse of health data seems different from the appropriate response to a failure of reliability. If a normally reliable person fails to come through for me, I might be upset or frustrated, but I should not blame the person for failing to come through because they had no obligation to me to do so. They simply failed to act as I predicted they would. Conversely, if a commercial company misuses my data, it does seem appropriate to place blame, in addition to being upset or frustrated. Sanctions or punishments would be appropriate, where they would not in the case of failed reliability. This suggests an expectation greater than mere reliability.

### Confidence in commercial companies

I have argued that we should not base the sharing of health data with commercial companies on trust, or mere reliance. Instead, we should share data with companies when we can do so with confidence. This means that sharing health data with commercial companies must take place within a system that is worthy of confidence; a system that assures the appropriate behaviour of commercial companies through laws and regulations, and does not rely on their motives or commitments. Within such a confidence-worthy system, individuals can predict how companies will use their data, and have assurances that it will be used appropriately.

This is not merely an argument for changing the way we talk about sharing data with commercial companies. Having confidence in an interaction is quite different from placing trust in another person or group. Replacing talk of trust with that of confidence implies a different set of expectations for both commercial companies using data, and individuals making it available to them.

There are advantages to pursuing a confidence-worthy system of data sharing with commercial companies, rather than one based on trust. As discussed earlier, confidence is more appropriate than trust when we require assurances or guarantees of certain behaviour. The potentially conflicting motives or commitments of commercial companies using health data justifies this need for assurances. Further, while it is debatable whether commercial companies have a moral obligation to be trustworthy, they do have a legal obligation to adhere to the laws and regulations that structure the systems in which they operate. Accordingly, the obligation to behave according to the laws and regulations of a confidence-worthy system is an enforceable obligation.

One way of assuring the appropriate use of health data by commercial companies is by imposing certain conditions on its use, and penalties in cases of data misuse. A confidence-worthy system can thus compel appropriate behaviour from commercial companies in a way that interactions based on trust cannot. Outside such a system, if a commercial company misuses an individual’s health data, there is very little that the individual can do. A confidence-worthy system can use incentives or sanctions to regulate behaviour in the absence of, or even contrary to, an individual or group’s motives or commitments. The ability to impose sanctions makes interactions predictable, insofar as both parties know that failing to live up to the terms of an agreement will have a particular result. The threat of sanctions might be useful as a deterrent for improper behaviour, as well as a means of recompense for those who are harmed or wronged.

The General Data Protection Regulations 2016 (GDPR)^29^ already imposes conditions on the use of personal data, including health data, and may issue substantial fines for violating these conditions. These fines may be fixed sums, or a percentage of a company’s annual revenue (which can result in harsher penalties for larger companies). They also include provisions for individual data subjects to take action against individuals or organisations that misuse their data. Additionally, regulations in the UK prevent the granting of exclusive licenses for the use of public-sector information (eg, NHS health data).^30^

In addition to these legal requirements, those sharing patient data (eg, databanks, NHS Trusts) might impose other discretionary requirements on its use by commercial companies. For example, they might restrict data sharing to only those activities that will benefit present or future patients, or even certain groups of patients (eg, specific illnesses). Contracts for sharing data with commercial companies might include provisions for those sharing data to seek damages in the event that the contract is breached. Because misuse of data can undermine public confidence in the system of data sharing more generally, it may be appropriate to impose harsher penalties on larger companies, insofar as their misbehaviour could be perceived as a greater threat to confidence. However, commercial companies looking to limit financial exposure may desire to minimise such discretionary requirements; a balance is needed between dissuading non-compliance, while also making data sharing sufficiently attractive.

The terms under which data are shared should be clearly described in a data-sharing agreement. This agreement should specify the purpose of the data sharing initiative, what data items will be shared and who will have access to them, and the security measures in place to protect the data along with procedures for dealing with potential data security incidents. Data users should be subject to regular audits by those sharing the data (or a designated third party), to ensure that they are compliant with the data-sharing agreement. In the event of non-compliance, access to the data should be suspended until the data processor is compliant or revoked permanently.

Of course, no system can rule out the possibility of so-called ‘bad actors’: those who do not adhere to the rules and regulations of the system. Accordingly, no realistic system of data sharing with commercial companies can eliminate the possibility of data misuse (although neither could one based on trust). Yet the mere possibility of data misuse need not undermine confidence in a system of data sharing as a whole. Different systems will require different degrees of reliability in order to be worthy of confidence (eg, public transit vs nuclear weapons). A system of sharing data with commercial companies can be designed to minimise the impact of bad actors, but this will require trade-offs: sharing data less freely might make it more secure, but result in fewer societal benefits. How these trade-offs should be managed in a confidence-worthy system will depend on its larger purpose. I return to this question below.

A confidence-worthy system of data sharing with commercial companies, rather than one based on trust, also has implications for how we think about consent. One of the challenges of sharing health data with commercial companies is that at the time the data are collected the details of how it will be used are unknown. This means that individuals sharing their data cannot be informed about the precise nature of the research in which their data will be involved. While it is possible to recontact individual data contributors to gain their informed consent each time their data are used, this would require re-identifying data that has been de-identified and could undermine participant confidentiality. It would also be highly burdensome for researchers and participants.

Several alternative models of consent have been proposed, including blanket consent, broad consent and dynamic consent.^31–33^ Roughly speaking, what distinguishes them is the degree to which the individual data contributor determines the specific uses of their data. Blanket consent effectively permits any and all uses of data once it has been provided, broad consent permits a range of future uses under a particular governance framework, and dynamic consent allows individuals to continually update their preferences for how data are shared. While I will not argue for any of these models of consent here, it is worth pointing out that each one involves varying degrees of confidence in the governance framework structuring how data are shared, and trust in the person or group making sharing decisions on behalf of the data contributor. The greater the decision-making power the individual data contributor delegates to the person or group sharing or using their data, the greater the role of trust, rather than confidence, in data-sharing decisions. Accordingly, the model of consent we adopt for sharing data with commercial companies should limit the discretionary power of commercial companies as much as required to ensure confidence. Further work is required to determine which of the existing models of consent best meets this requirement.

## Confidence-worthy health data sharing with commercial companies

I have argued that sharing data with commercial companies should be based on confidence, rather than trust. To achieve this, data sharing must take place within a confidence-worthy system. In the next section, I briefly describe four key features of a confidence-worthy system for sharing health data with commercial companies: transparency, accountability, representation and a clear purpose. The aim here is to provide a starting point for thinking about a system of data sharing with commercial companies that is worthy of confidence.

### Transparency

Confidence-worthy systems must be transparent. One cannot interact confidently within a system without a basic knowledge of the rules and norms that comprise it. Importantly, information availability does not constitute meaningful transparency. As Onora O’Neill argues, information availability can prevent secrecy, but not deception.^34^ A system that requires information be made freely available can encourage people to be less honest, or distort the truth. Unsorted information or misinformation can create confusion and uncertainty, undermining confidence in a system.

Meaningful transparency occurs when information is provided in a way that allows individuals to actively check its veracity, and assess the credibility and reliability of those conveying the information. Meaningful transparency also allows the system *itself* to be subject to public scrutiny, and to ensure that it is not vulnerable to manipulation. When a system fails to function properly, meaningful transparency helps to ensure that the cause can be identified and remedied, rather than ignored or hidden.

A system of sharing patient data with commercial companies that is meaningfully transparent will allow individuals to judge whether companies are using their data in a way that is consistent with the system’s rules and regulations. Individuals need not make this assessment themselves, although the necessary information should be available if they choose to do so. Data users could be required to agree to regular auditing by an independent third party, including providing access to the relevant facilities to review storage and security of the data. The results of these audits should be made available to the public on the data sharer’s and data user’s website. Applications for data access can be similarly published, as well as the criteria by which proposals for data sharing are accepted, and the vetting process used to determine whether a particular company is given access to patient data.

### Accountability

While transparency ensures that the rules and norms governing a system are accessible, accountability helps to ensure adherence to these rules and norms. Sanctions contribute to the confidence-worthiness of a system by helping to define and enforce the boundaries of permissible behaviour. Yet poorly chosen instruments of accountability can sometimes obstruct the proper functions of an institution or system, which can in turn undermine confidence. The means of accountability should reflect the true aims of a system, and not merely what is easily measurable. For example, a central aim of sharing health data with commercial companies is to ‘improve the health, welfare, and/or care of patients’.^12^ Reducing this complex aim to a simple metric such as reduced wait times in hospital, or improvements in quality adjusted life years per dollar spent, may incentivise companies to produce tools that target these particular measures, but which may or may not actually achieve the real goal of improving health and welfare.

Because the aims of a system of sharing patient data with commercial companies are complex, not everything is reducible to a simple procedure. The way companies are held accountable must reflect this complexity. Ensuring accountability may not lend itself to a series of stock performance indicators or ‘box-checking’ exercises, but require companies to provide a more nuanced account of how they are using patient data to independent parties that have the experience and competence to assess it. The purpose of accountability is not to ensure that arbitrary performance indicators are met, but to render a substantive, fair, and independent judgement of an institution’s work. Ensuring that companies are accountable will require those tasked with ensuring accountability some room for interpretation. For example, health and care organisations in the UK must appoint a ‘Caldicott Guardian’, a senior person responsible for ensuring the appropriate use of personal information. Caldicott Guardians are guided by a set of principles,^35^ but must also exercise judgement in applying them. A similar independent monitor could hold companies accountable in their use of health data, guided by principles but also using their own judgement.

### Representation

A system cannot be worthy of confidence if it provides reliable outcomes for some but not others. Accordingly, it must be representative of those interacting within the system, such that it functions well for all users. This is of particular importance for a system of sharing health data with commercial companies, given the inequalities that already exist within the wider health system, and their potential impact on the development of data-driven digital health technologies. For example, research has shown that there are systematic differences in the quality and quantity of health data, leading to datasets that under-represent key segments of the overall population.^36 37^ When used to develop and validate digital health technologies, these datasets can lead to interventions that are safe and effective for some, but ineffective and even dangerous for others.^38^

Ensuring that a system of sharing health data with commercial companies is representative is no easy task, requiring engagement at various levels of the system. Regulators, policy makers and politicians must impose and enforce rules to ensure that the digital health tools developed by companies are effective across different populations and contexts. Steps must be taken to engage with the public—particularly those who are under-represented in health data—about the importance of their health data in developing health technologies that are safe, effective and equitable, while also providing assurances that reflect their concerns about data sharing. Investment is needed to build datasets that are sufficiently representative of the population in which digital health technologies will be deployed, and of sufficient quality and quantity to validate the safety and efficacy of these technologies. Investment is also needed to provide equitable access to digital technologies, both for the purposes of gathering data, and providing patient benefit.

Public involvement should be an ongoing part of any large-scale initiative for sharing health data with commercial companies. This might take the form of participant representation on data access committees and governance committees, or independent public advisory boards. Engaging the public during the planning stages of data sharing initiatives will help to establish legitimacy and foster confidence. This includes public consultation on the consent model for data collection and sharing, as well as models for capturing the plurality of ways in which health data are valuable.^39^ Different models for deliberative approaches to biobanks have been used successfully, and could be adapted to the sharing of health data.^40^

### Social purpose

In addition to accountability, transparency, and representation, a confidence-worthy system requires a defined purpose, based on the aims or goals of the parties involved: what is the purpose of the interaction that the system is meant to structure?

One way of framing this issue is to ask what purpose commercial companies using health data should serve within the larger social systems in which they operate. As Hsieh and colleagues argue, this is a matter of the ‘social purpose’ of commercial companies: the specific contributions a company makes to advancing societal goals.^41^

On some accounts, pursuing financial objectives guides companies to their true social purpose—making efficient use of societal resources.^42^ Such a view assumes that commercial companies fulfil their social purpose simply by maximising profits. However, there is a wider range of social purposes that a company might pursue.^41^ Moreover, commercial companies are embedded within larger political and social institutions, which arguably ought to have some influence on their social responsibilities.^41^

Another way of framing this issue is in terms of the social role of individuals within the health system. In addition to being the beneficiaries of care, research has found that people in the UK also identify behaviours like reciprocity, altruism, and solidarity as a part of their role within the health system.^43^ Conversely, when one interacts with a company or another individual and occupies the role of ‘customer’ or ‘consumer’ (or even ‘product’), the norms which structure this interaction are different. Fundamentally, these norms enable mutually self-interested cooperation. There is no expectation that I behave altruistically in my interactions with a commercial company, for example, nor is there any expectation that they will act in my best interests.

Sharing health data with commercial companies imposes new kinds of obligations and expectations on individuals to contribute to the functioning of the health system. Contributing to commercial research does not obviously fall within the existing norms that comprise the patient role (ie, reciprocity, altruism, solidarity).^2^ Some commentators have argued in favour of revising the patient role in this way.^44^ Whether or not one supports a healthcare model that embeds research in practice, however, a re-evaluation of the role of ‘patient’ to include on obligation to contribute to healthcare learning (including by sharing data with commercial companies) requires a commensurate re-evaluation of the structures in place to protect their interests.

Changing the patient role can also influence the nature of existing interactions. For example, if patients are worried that their health data are being used in ways that are not in their best interests, they may be less willing to share this information with their physician. Making data available to commercial companies also increases the possibility of a privacy breach or data misuse, which patients may construe as an unnecessary risk that is inconsistent with their interests.

Changing the patient role also introduces new ‘interactions’ between patients and commercial companies. While companies and patients are unlikely to deal with one another directly, the use of their data by commercial companies can nevertheless affect patient interests. What is the appropriate orientation for patients to adopt, when choosing whether to share their data: are they ‘patients’ or are they ‘products’?. Can they reasonably expect that companies using their data will abide by the relevant regulations, but are otherwise self-interested? Alternatively, can patients reasonably expect that due consideration will be given to their interests, or to broader societal benefit?

How we understand the social purpose of the activities in which these companies engage should influence the design of the system of data sharing we adopt. If we consider the practice of commercial health research as akin to a contract between mutually disinterested parties (eg, in which we as a society want new technologies, and companies want to make money), our system can reflect this arrangement. In this case, the system would require clear terms for how data may permissibly be used, and appropriate sanctions applied in the event of non-compliance, but could otherwise allow companies to use patient data in whatever way is most profitable. Conversely, if we consider the research enterprise to be more than just a contract between mutually disinterested parties, (eg, if we view health research as reflecting broader societal goals and values about what we think is worth pursuing), our system of sharing patient data ought to reflect these values. Here, we might require the use of patient health data to be restricted to research that promotes broader societal interests (eg, health, social, environmental), even at the expense of commerical gains.

There is likely to be a range of views regarding the social purpose of a system of health data sharing in general, and the involvement of commercial companies in particular. In this respect, the concept of ‘data trusts’ might provide a useful model for data sharing with commercial companies.^45^ In this model, a trustee (eg, a data controller) stewards the data on behalf of the data beneficiaries. Different data trusts can set different terms for data management, according to the views of their beneficiaries.^46^ Insofar as they provide a mechanism for stakeholders to provide input to how data are used, shared and managed, as well as on the core assumptions and motivations of the data trust itself, these trusts are a useful means to building the confidence of data subjects in sharing their data.

## Role of trust in a system of health data sharing

I have argued to this point that sharing health data with commercial companies should be based on confidence rather than trust. By restricting my argument to commercial companies, however, I have not argued that there is no role for trust in a system of sharing health data. Indeed, there is likely to be a significant role for trust in this larger system, but commercial companies should not be its object.

On the one hand, it might be important that certain individuals or groups have the right kinds of motives or commitments. Within a system of sharing health data, for example, we might think it is the responsibility of physicians and health institutions to act *for*, and not merely consistent with, patient interests.

On the other hand, in many systems, it is not only desirable but also necessary to leave room for self-governance. Complex activities may not reducible to following a set of rules or procedures. Our system should be sufficiently fine-grained to ensure the outcomes that we as a society want from data sharing. As mentioned earlier, this should include limits on the purposes for which health data may be used (eg, health, social, environmental benefit, with public input into what constitutes such a benefit), and the necessary measures that must be taken to ensure data security and quality. It might also include regulations on the price that commercial companies can charge for products resulting from research using public data, to ensure that these technologies are accessible, or provide incentives for the development of innovative rather than iterative products. Within the space marked out by these rules and regulations, companies can then pursue their own aims (eg, profitability).

Of course, in some cases, how the rules of the system are interpreted matters. For example, a possible requirement of companies using health data to design algorithms might be that they train and validate them using datasets that are representative of the population in which they will be deployed. Whether a dataset qualifies as representative for a particular context may not be specifiable ahead of time, and so cannot be written into regulations. Leaving this requirement open to interpretation requires placing trust in whomever is doing the interpreting. Accordingly, it should not be done by companies but by independent regulators whose motives and commitments (ie, the basis of their trustworthiness) are aligned with the interests of the public.

## Conclusion

Commercial companies will continue to play a central role in health research. Their involvement brings with it great promise for advances in treatment and care, but also requires vigilance. I have argued that rather than placing our trust in commercial companies to use health data appropriately, we should implement a system for sharing health data that is worthy of confidence. My purpose in this paper has been to defend a normative account of our orientation to commercial companies with respect to sharing patient data (and some of the features of such an orientation), rather than argue for a particular regulatory framework. Careful thought is needed to fill in the details of such a framework, and will require a combination of normative and empirical work. Indeed, as research moves forward and new applications for health data are devised, the system of data sharing may need to evolve as well, to allow us to continue to share our data with confidence.

## Footnotes

While my understanding of confidence draws from the work of Luhmann, Simmel and Giddens, my use of the concept should not be understood as a strict interpretation of their work.

In the UK, patients have the option of opting out of sharing their health data for research and planning purposes, meaning that sharing data with commercial companies is not a requirement of the patient role. However, making data sharing the default option not only makes people more likely to select it, it also tacitly conveys that data sharing is an expectation.
